# Application of ZnO-Nd Nano-Photocatalyst for the Reactive Red 198 Dye Decolorization in the Falling-Film Photocatalytic Reactor

**DOI:** 10.3390/toxics9100254

**Published:** 2021-10-08

**Authors:** Fatemeh Biglar, Amirreza Talaiekhozani, Farham Aminsharei, Junboum Park, Anahita Barghi, Shahabaldin Rezania

**Affiliations:** 1 Faculty of Chemical, Petroleum and Gas Engineering, Semnan University, Semnan 35131-19111, Iran; f.biglar1991@gmail.com; 2 Department of Civil Engineering, Jami Institute of Technology, Isfahan 84919-63395, Iran; 3 Department of Safety, Health and Environment, Najafabad Branch, Islamic Azad University, Najafabad 85141-43131, Iran; aminsharei.fa@gmail.com; 4 Human Environment and Sustainable Development Research Center, Najafabad Branch, Islamic Azad University, Najafabad 85141-43131, Iran; 5 Department of Civil and Environmental Engineering, Seoul National University, Seoul 08826, Korea; junbpark@snu.ac.kr; 6 Division of Environmental Science and Engineering, Pohang University of Science and Technology (POSTECH), 77 Cheongam-ro, Pohang 37673, Korea; anahitabp@postech.ac.kr; 7 Department of Environment and Energy, Sejong University, Seoul 05006, Korea

**Keywords:** ZnO-Nd nano-photocatalyst, photocatalytic degradation, falling-film photo reactor, reactive red 198, RSM

## Abstract

A large amount of Reactive red 198 (RR198) is released yearly into the environment. RR198 is toxic for human and aquatic creatures; therefore, it should be removed from wastewater before releasing into the environment. In this study, the nano ZnO-Nd -photo-catalyst for the first time was synthesized by the combustion method. First, the physical characteristics of the generated nano photocatalyst were evaluated using FESEM, XRD, Bandgap calculation, and FTIR analysis. Then, the ZnO-Nd nano-photocatalyst was suspended into the contaminated water with RR198 dye in a falling-film photocatalytic reactor. The effects of parameters such as the amount of H_2_O_2_, catalyst dose, pH, and initial concentration of dye were investigated during the experiments. Finally, the decolorization process with the falling-film photocatalytic reactor was optimized using response surface methodology (RSM). The physical characteristics showed that the average particle size of the synthesized ZnO-Nd was 40 nm. Doping ZnO with Nd reduced the photocatalyst energy bandgap by 14%. The results indicated that the optimum amount of catalyst dose and pH level was 0.1 g/L and 5, respectively. The simultaneous usage of H_2_O_2_ and ZnO-Nd with an H_2_O_2_/dye ratio of two increased dye removal performance by 90%. The results demonstrated that the developed equations can be applied to predict the performance of the falling-film photoreactor. This study showed that using the nano ZnO-Nd photocatalyst in a falling-film photocatalytic reactor under optimum operating conditions is an appropriate way to remove RR198 from water.

## 1. Introduction

Discharging wastewater from cities, especially large cities and industries, is considered to be the main reason for surface water and groundwater pollution [1]. Nowadays, more than half of the global population lives in cities, and recently published papers illustrate that the number of people who are affected by organic pollution will dramatically increase from 1.2 billion in 2000 to 2.5 billion in 2050 [2]. Every day, thousands of various dyes are used in different industries [3,4]. Generally, the used dyes in the industries can be released into the environment via their wastewater. Therefore, finding an appropriate way to remove such dyes from wastewater is important [5]. Azo dyes are one of the most important dyes and RR198 is one of the most common azo dyes that is used in industries. Since this dye has stable chemical properties and structure, it is difficult to degrade under natural conditions [6].

The wastewater of textile, paper, and some other industries contains dyes. Physical, chemical, and biological methods have been utilized to eliminate dyes from various wastewater. A physical method only transfers the dye from the liquid phase to the solid. This creates secondary contamination which requires more filtration. Biological processes are common methods for the filtration of industrial wastewaters due to their low cost [7]. On the other hand, azo dyes with one or more N=N bonds account for 60–70% of all dyes. Azo dye molecular structures are complex; therefore, degradation of such dyes under aerobic biological conditions does not have an appropriate performance. The anaerobic biological degradation has been reported as the most effective biological process to remove azo dyes [8]. Anaerobic biological processes are complex and face many difficulties, such as the production of malodorous compounds [9].

Advanced oxidization processes (AOP) have attracted much attention in the last two decades as a wastewater filtration method. These processes successfully destroy organic contaminants on a laboratory scale. AOP includes methods such as ozonation, ultraviolet (UV)/H_2_O_2_, Fenton, photo-Fenton, and photocatalytic oxidation processes [10]. Although these processes are used in various reaction systems, they have similar chemical specifications. Their main similarity is the application of highly reactive oxidizing agents, such as radical hydroxyl (OH^o^) [11,12]. During the process, AOP can produce reactive hydroxyl radicals (OH^o^) with organic compounds and reduce the oxidation potential up to 2.80 eV [13]. This amount is more than the oxidation potential of radicals of sulfate, chlorine, permanganate, persulfate enzyme, hydrogen peroxide (H_2_O_2_), and ozone (O_3_). However, the oxidation potential of hydroxyl radicals is less than Florine (3.03 eV). Many reports have illustrated that the photocatalytic processes show a good capability in dye degradation in water [14,15]. They have an extensive application due to their good performance in liquid and gaseous systems [5,16]. ZnO and TiO_2_ are the most used photocatalysts in various studies. These metal oxides perform well and they can destroy complex molecules which common methods cannot destroy [17]. Many studies have illustrated that using ZnO and TiO_2_ at the nano size can increase their performance in removing environmental pollutants [16]. Although nanoparticles have a vast variety of applications in biology, medicine, engineering, environmental sciences, etc. [18,19], many studies have shown that some nanoparticles in high concentrations can be harmful to human and animal health [16,20]. Therefore, such nano-catalysts must be used under certain concentrations to avoid negative effects on human and animal health. The nano-catalysts can destroy contaminants to form H_2_O and CO_2_ [21]. Some reports suggest that ZnO under certain circumstances can be more efficient than TiO_2_ [16]. The ZnO is one semiconductor that has a wide bandgap of 3.37 eV in the wurtzite phase, resulting in a further limitation in its application [22]. Some studies on decreasing the bandgap of nanocomposites showed that better electron-hole separation and higher efficiency are obtained with a narrower bandgap [23,24]. Modification of ZnO nanoparticles is conducted by doping or replacing them with certain atoms. This method improves its electrical and optical properties by changing surface properties [25]. There are many various methods to synthesize ZnO and TiO_2_ nanoparticles, and among them the well-known and most used method is the sol-gel method. However, these days more efficient methods, such as the combustion method, have been introduced for the synthesis of nanoparticles.

The combustion synthesis method is one of the most effective methods for various materials production such as advanced ceramics, catalysts, composites, and nanomaterials. This method uses a reduction-oxidation (redox) reaction to produce the energy required instead of flame. Recently, nanoparticles obtained by this method have drawn interest and a few researchers have used this method for the production of photocatalysts [26].

Although several studies have been done on photocatalytic dye removal, the RR198 dye removal in a falling-film photocatalytic reactor using ZnO-Nd nano-catalysts synthesized by the combustion method has not been investigated. This study aims to determine the optimum amount of H_2_O_2_, catalyst dose, pH, and initial concentration of dye required, as well as to develop an equation to predict the performance of falling-film photoreactor-containing ZnO-Nd nano-catalysts under different parametrical conditions.

## 2. Materials and Methods

### 2.1. Materials

The mono-azo RR198 dye was purchased from Alvan Sabet Corporation, Tehran, Iran. The λ_max_ related to the dye was 520 nm. The high purity zinc nitrate and neodymium dopant were purchased from Merck, Darmstadt, Germany. Deionized water with a conductivity of 0.8 µs cm^−1^ was used. Other materials such as sulfuric acid, sodium hydroxide, etc. were supplied from Merck. The chemical formulation of RR198 is C27H18ClN7Na4O16S5 and it has a complex structure with the five benzene rings which contain double-bonded nitrogen [27].

### 2.2. Synthetic Wastewater

In total, 150 mg of RR198 dye powder was weighed by a digital balance and then added to the 750 mL of distilled water to obtain a dye solution with 20 ppm. During the experiments, acidic and alkaline synthetic wastewater were tested to find the optimum pH. The pH value of the solution was adjusted by the addition of a suitable amount of 0.2 N sulfuric acid solution or a hydroxide sodium solution. Sulfuric acid and hydroxide sodium were used when acidic and alkaline synthetic wastewater was needed, respectively.

### 2.3. ZnO:Nd Nanoparticle Synthesis

Zinc oxide nano-catalyst and neodymium were combined by the solution combustion method to synthesize ZnO-Nd. To do this, 5 g of zinc nitrate, 1.15 g of glucose (as fuel), 0.1 g of neodymium nitrate, and 0.1 g of glycine (as a fuel aid) was added to 20 mL of deionized water. The sample was then heated to 80 °C to form a yellow gel. Then, the obtained gel was placed under the 900 W microwave (Samsung, MS23F300EEW made in Korea) for 1 min to ignite the sample. To remove the residual gases and carbon from the combustion process, the synthesized foam was then heated to a temperature of 500 °C. The temperature was gradually increased at a rate of 10 °C per min. The foam was heated at 500 °C for one hour. Eventually, the color of the nano-catalyst turned to white. Then the synthesized ZnO-Nd nano photocatalyst was characterized by different methods. The X-RD was used for phase identification of a crystalline material and to provide information on unit cell dimensions. The surface morphology of the synthesized ZnO-Nd nano-photocatalyst was investigated by the FE-SEM. FT-IR was also used to investigate the chemical structure of synthesized ZnO-Nd nano-photocatalyst.

### 2.4. Experimental Setup

**Figure 1:**
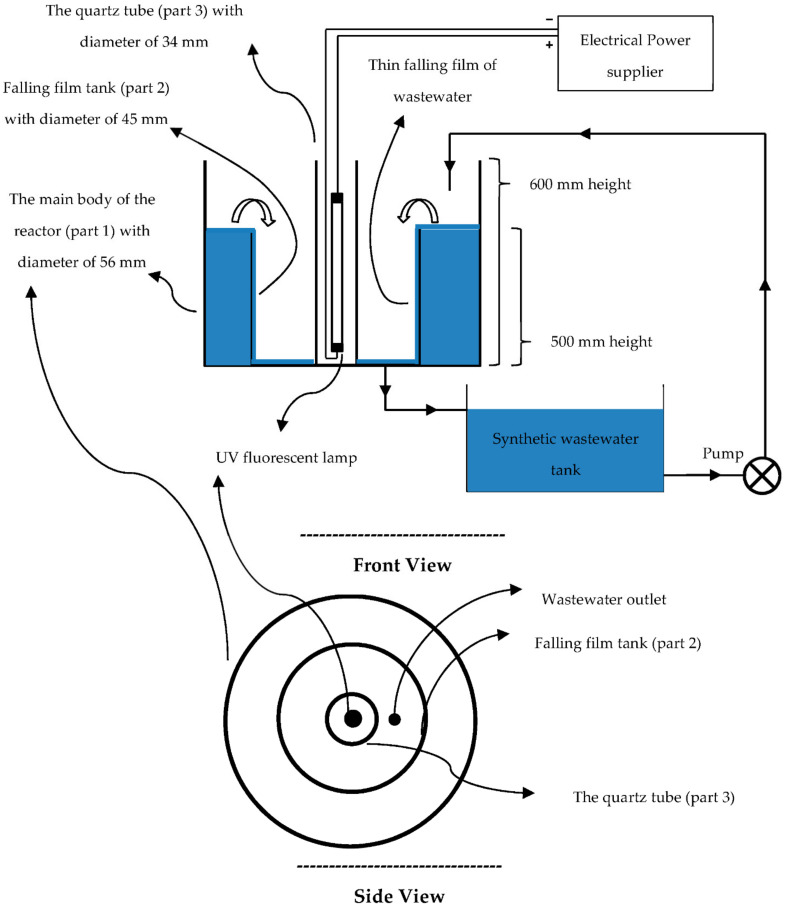
Schematic diagram of the falling-film photo reactor used for degradation of RR198.

In this study, a falling-film photo reactor was designed, which is shown in Figure 1. Falling-film reactors provide a thin synthetic wastewater film, thus it generates low absorption of UV light especially in dark colors so dye degradation increases [28]. Most of the falling-film photo reactors are continuous, while a semi-batch photo reactor was designed in this study. In the designed photo reactor, the water contaminated by RR198 can be circulated many times; therefore, it does not require too much height or pump head.

The falling-film photo reactor was divided into three separate cylindrical parts. The first part was the main body where all other reactor parts were installed. The diameter of the main body was 56 mm, and the height was 60 cm. The initial volumetric flow rate of contaminated wastewater was 21.63 mL/s. The second part of the photo reactor was the falling-film tank, with a diameter of 45 mm, a height of 500 mm, and a volume of 225 mL. The third part of the photo reactor was the quarts tube, with a diameter of 34 mm and a height of 600 mm (Figure 1). UV OSRAM G13 (15W) UVC lamp made in Iran with 15 W power was located inside the quartz tube. The wavelength of emitted UV light from the lamp was between 200–280 µm. UV radiation could easily transfer from the quarts tube and reach the falling-film part; therefore, suspended ZnO:Nd nanoparticles would be under UV radiation easily. The synthetic wastewater contaminated with RR198 was stored in a storage tank (see Figure 1). The storage tank was connected to the inlet of the photo reactor, as shown in Figure 1. The synthetic wastewater was pumped from the storage tank to the first part of the photo reactor. Because the pump was running continuously, the first part of the reactor was filled with synthetic wastewater, and eventually the wastewater slid down to the walls of the third part of the photo reactor to form a thin falling film of synthetic wastewater (see Figure 1). The thin film was close to the quarts tube, which was exposed to UV radiation. Falling-film reactors provide a thin synthetic wastewater film, thus UV can fully penetrate the wastewater to reach the suspended ZnO:Nd nanoparticles. In this condition, the dissolved RR198 dye in wastewater and the photocatalysts were under UV radiation and in contact with each other. Therefore, photocatalytic reactions happened, and the dye was removed from the wastewater. The thickness of the falling film was approximately 1 mm. In addition, the height of the second part of the photo reactor was 50 cm and the volume of falling film of the synthetic wastewater was around 5 mL. The volume of the thin falling film can be considered as the active section of the reactor volume. In each experiment, a suitable amount of ZnO:Nd nanoparticles were added to the synthetic wastewater tank to obtain catalyst concentrations of 40, 60, 80, 100, and 120 mg/L. Then, the pump was turned on to start the experiment. Before and after each experiment, the dye concentration was measured using the total organic carbon (TOC) analyzer. The performance of dye removal was calculated using Equation (1).

(1) $$\mathrm{RE}=\frac{C_{\mathrm{in}}-C_{\mathrm{out}}}{C_{\mathrm{in}}}\times 100$$

where RE is dye removal efficiency (%), C_in_ is the initial dye concentration (mg/L), and C_out_ is the dye concentration after the photocatalytic degradation (mg/L).

### 2.5. Effect of ZnO-Nd Nanoparticles Concentration

One of the parameters affecting the dye removal performance is the amount of photocatalyst used. The aim of this section is to determine the photolysis contribution in the dye destruction and the minimum catalyst intake for maximum decolorization efficiency. First, the experiment was carried out in the absence of a photocatalyst and then the performance of dye removal was determined. To obtain the highest performance of photocatalyst, different concentrations of the ZnO-Nd nanoparticles (0.04, 0.08, 0.1, and 0.12 g/L) under various pH values (5 and 10) with a dye concentration of 20 ppm in the absence of hydrogen peroxide were used.

### 2.6. Effect of pH

The effect of pH on the photocatalytic activity of ZnO-Nd nano-photocatalyst was investigated by adjusting the synthetic wastewater pH on 3, 5, 7, and 9. The experiments were done with a dye concentration of 20 ppm and a catalyst dosage of 0.10 g/L. Each of the abovementioned experiments was repeated in four various HRTs (30, 60, 90, and 120 min).

### 2.7. Effect of H_2_O_2_

Hydroxyl radical (OH^o^) is a powerful and well-known oxidizer that has been widely used in various wastewater treatment studies. Since H_2_O_2_ can generate OH^o^ under UV radiation, it increases the RR198 dye removal from synthetic wastewater. Therefore, the presence of different concentrations of H_2_O_2_ in the falling-film photocatalytic reactor was investigated. Different ratios of H_2_O_2_ on dye concentration (H) were produced and tested in the experiments to find the effect of H_2_O_2_ concentration on dye removal from the synthetic wastewater. Four separate experiments were carried out by the falling-film photocatalytic reactor under dye concentration of 20 ppm, catalyst dosage 0.10 g/L, hydraulic retention time (HRT) of 120 min, and pH of 5 to investigate the effect of various H ratios.

### 2.8. Effect of the Initial Dye Concentration

The initial dye concentration has an important effect on the performance of the falling-film photocatalytic reactor to remove RR198 dye from synthetic wastewater. In this section, three experiments were done with the catalyst dosage of 0.10 g/L, pH of 5, HRT of 120 min, and H of 2. The initial dye concentration in the three abovementioned experiments was 10, 20, and 30 ppm, respectively.

### 2.9. Analytical Methods

**Figure 2:**
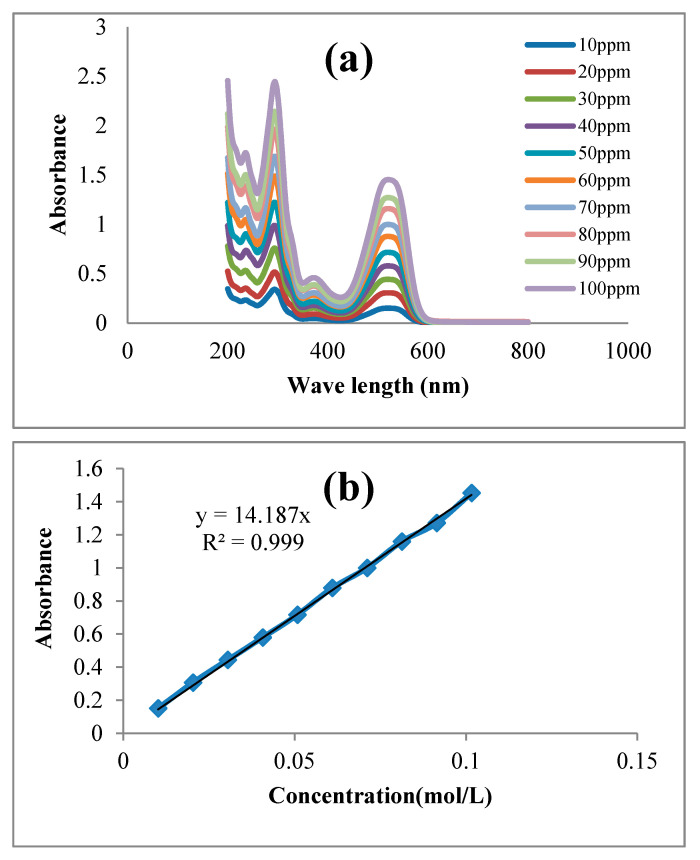
(**a**) The absorption of synthetic wastewater in various dye concentrations from 10 to 100 ppm between wavelengths of 200 and 800 µm, and (**b**) the relation between adsorption and the dye concentration.

X-ray powder diffraction (X-RD) analyses were performed using the Stadi P model of the X-ray diffraction device (STOE, Darmstadt, Germany). ZnO-Nd nano-photocatalyst surface morphology was investigated using a field emission scanning electron microscope (FESEM) using a Sigma VP-500 ZEISS company, Germany. The Fourier-transform infrared spectroscopy (FT-IR) tests were done by Perkin-Elmer One FTIR, Waltham, MA, USA. The TOC analyzer (Shimadzu, Kyoto, Japan) was used to check the rate of mineralization of the dye in the photo reactor over time. In this study, the concentration of dye was measured by a spectrophotometer (Shimadzu UV–1800) at a wavelength of 520 µm. Then, synthetic wastewater with a concentration of 10, 20, 30, 40, 50, 60, 70, 80, 90, and 100 ppm was prepared. The amount of absorption was measured by a spectrophotometer in wavelengths between 200 and 800 (Figure 2). Next, Equation (2) was calculated by a regression among the measured absorption in the synthetic wastewater. In this equation, A is the amount of absorption and C is the concentration of dye in the synthetic wastewater in mol/L.

A = 14187C (R^2^ = 0.99) (2)

The bandgap value was obtained with a UV/visible spectrophotometer (Perkin-Elmer lambda 25), as reported by Chen and Jaramillo [29]. The device was set to achieve a transmission curve and showed a T% -λ plot. Then Equations (3) and (4) were used to calculate a and hv.

(3) $$\alpha =\ln\left(\frac{1}{T}\right)$$

(4) $$\mathrm{hv}=\frac{1240.71}{\lambda }$$

then (a×hυ)^2^ was calculated and plotted by hv. The point at which the dip line crosses the x-axis is the bandgap. The pH value was measured by using a Mettler Toledo digital pH meter.

## 3. Results and Discussion

### 3.1. Photocatalyst Characterization

#### 3.1.1. X-ray Diffraction

**Figure 3:**
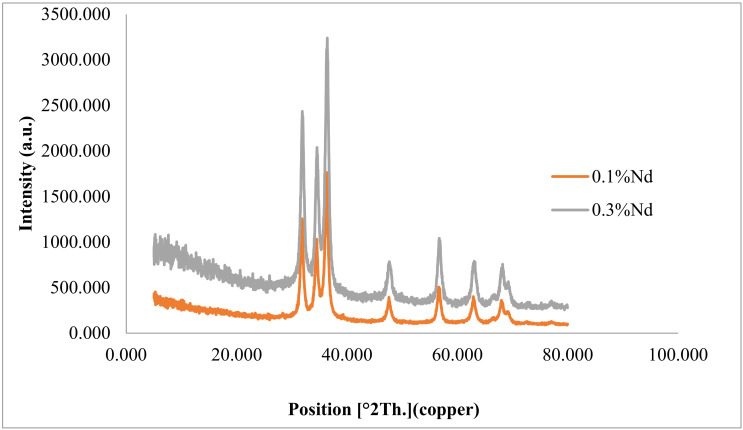
XRD patterns of ZnO doped with different values of neodymium nitrate.

The pattern for 0.1 and 0.3 gr of ZnO-Nd nanoparticles and neodymium nitrate is demonstrated in Figure 3. The diffraction peaks of 31.845, 34.523, 36.291, 47.601, 56.623, 62.889, 66.60, 67.985, and 69.103 show the crystalline nature with the peaks of 002, 100, 101, 110, 102, 103, 200, 112, and 201. These peaks are the crystal characteristics of hexagonal wurtzite [30,31]. They are detected based on space group p63mc, JCPDS data card no: 36–1451. The calculation of lattice constants with the parameter “a” was obtained by using the <100> plane and Equation (5).

(5) $$a=\frac{\lambda }{\sqrt{3}\sin\theta }$$

By using the <002> plane, the network constant, “c”, was obtained with Equation (6).

(6) $$c=\frac{\lambda }{\sin\theta }$$

The photocatalyst constants, including a = 3.2409 and c = 5.189, were calculated. ZnO network constants were a = 3.2299 and c = 5.1755 [32]. The change from pure ZnO network constant with doped Nd to ZnO nanoparticles can be attributed to the bigger ionic radius of Nd^3+^. The noteworthy point is that the photocatalyst sample creates no extra phase in comparison with the pure zinc oxide. The ionic radius of Zn^2+^ and Nd^3+^ were 0.0740 nm and 0.0983 nm, respectively. When the Nd^3+^ sites were replaced with Zn^2+^ sites, the values of d changed [25].

d 100 ZnO = 2.79717 Å; d 002 ZnO = 2.58775 Å d 100 ZnO-Nd = 2.81089 Å; d 002 ZnO-Nd = 2.60122 Å

The size of the crystal network was computed by Scherrer’s equation. These calculations were conducted for ZnO and 0.1 and 0.3 g neodymium nitrate-doped ZnO nanoparticles. Their sizes were 40.8 nm, 54.2, and 135 nm, respectively. This proves that the increase in the size of crystals is constant [30].

**Figure 4:**
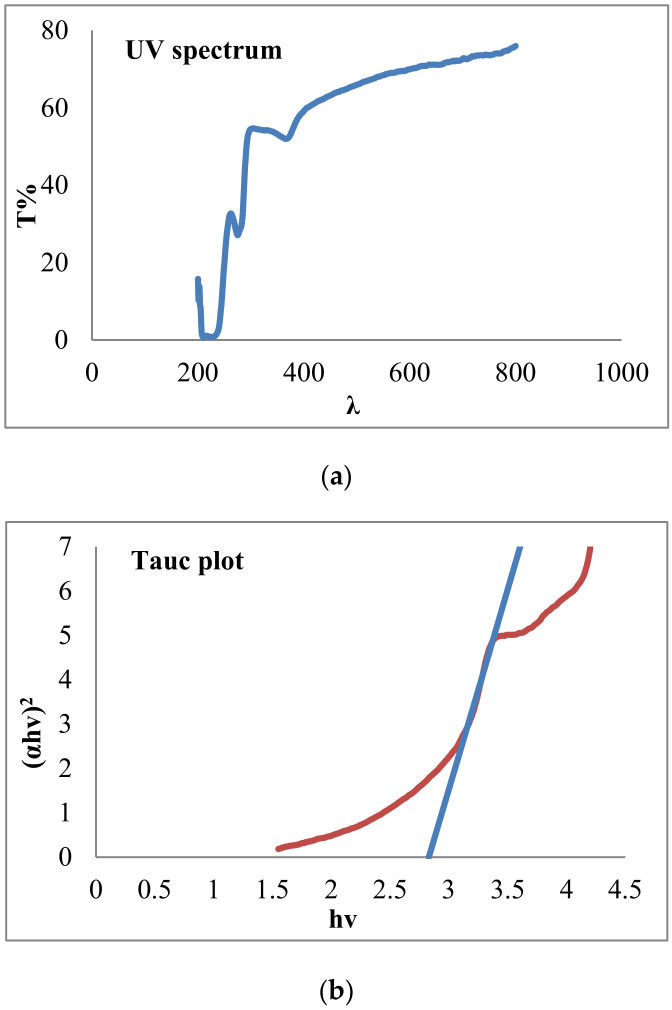
(**a**) The results of UV-Vis spectra and (**b**) Tauc plot for bandgap energy of ZnO-Nd.

Bandgap value was obtained with a Perkin-Elmer lambda 25 UV-Vis spectrophotometer device (Figure 4a) and the Tauc plot method. Equation (7) was also used:

(7) $$A\;h\upsilon ={\;A}_{0}\;{\left(h\upsilon -{\;E}_{g}\right)}^{2}$$

where a is the absorption coefficient, and hν and E_g_ are the photon energy and bandgap energy, respectively. Both measurements are in eVs. A_0_ and 2 are the constants that depend on the type of electron transition [23]. As Figure 4b shows, the bandgap reduced from 3.3 to 2.89 electron volts. This result indicated that the optical property of ZnO improved with doping neodymium nitrate.

#### 3.1.2. Scanning Electron Micrograph

**Figure 5:**
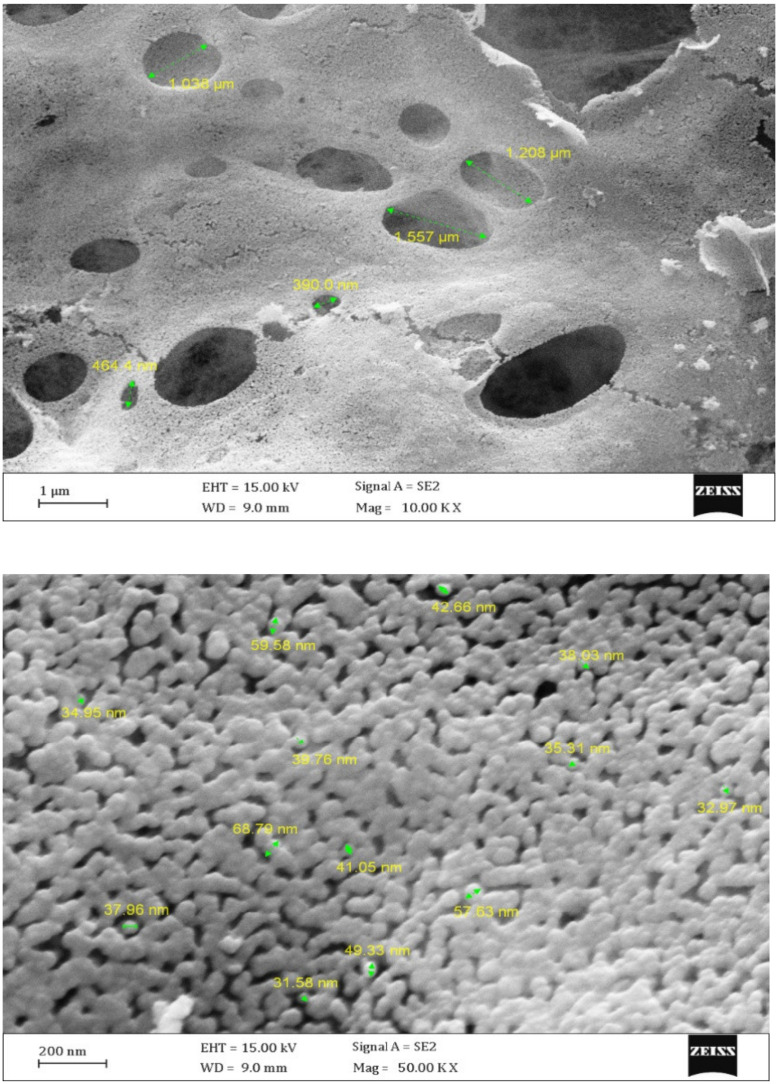
FESEM images of ZnO-Nd photocatalysts.

The SEM images proved that the particles were small enough to say that they were nanoparticles, and the crystal sizes were between 31.58 and 68.79 nm. Moreover, Figure 5 shows that the dimensions of created porosity had a maximum size of 1.557 micrometers. Based on the results, the nanoparticles contain porosities of different sizes. This can be related to the type of combustion synthesis and exhaust gases resulting from combustion, such as carbon dioxide and nitrogen dioxide. The shapes of nanoparticles are spherical and cubic.

#### 3.1.3. Fourier-Transform Infrared Spectroscopy

**Figure 6:**
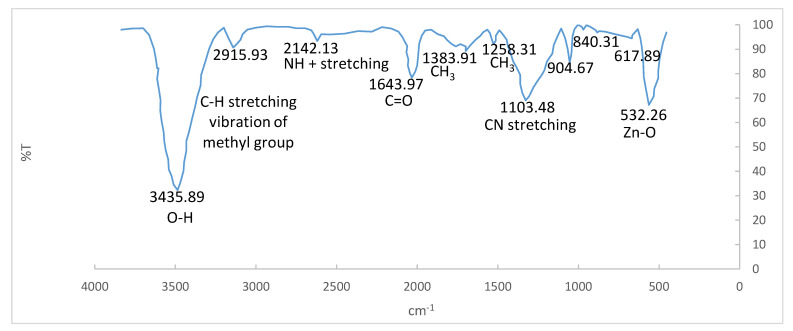
FTIR spectra of the ZnO-Nd photocatalyst.

The ZnO-Nd photocatalysts produced by the combustion method were investigated by FTIR. In this research, a range of 450 to 4000 cm^−1^ was studied. As shown in Figure 6, peaks can be seen at 3435.89, 2142.13, 1634.97, 1383.91, 1258.31, 1103.48, 904.67, 840.31, 617.89, and 532.26 cm^−1^ and shown in Figure 6. The peak of 532.26 is for the metallic bond of oxygen and zinc (Zn-O) [33,34]. The peaks of 3435.89 and 2918.93 cm^−1^ are related to O-H and C-H stretching vibration of methyl groups. The peak of 1634.97 cm^−1^ is also related to C=O. The peak of 1103.48 is for C-N stretching, and 1383.91 cm^−1^ and 2142.13 cm^−1^ peaks are related to CH_3_ and NH^+^ stretching, respectively [35].

### 3.2. Photocatalyst Experiments

#### 3.2.1. Effect of Amount of ZnO-Nd

The removal of certain dyes by UV radiation alone has been reported [36,37]. This means that a significant amount of some dyes, such as acid orange 7 dye, can be removed from water under UV radiation [38]. Therefore, an experiment was planned to ensure that a significant amount of the RR198 dye could not be removed under the irradiation of UV. The results showed that only 6.49% of RR198 dye was removed by UV radiation alone.

**Figure 7:**
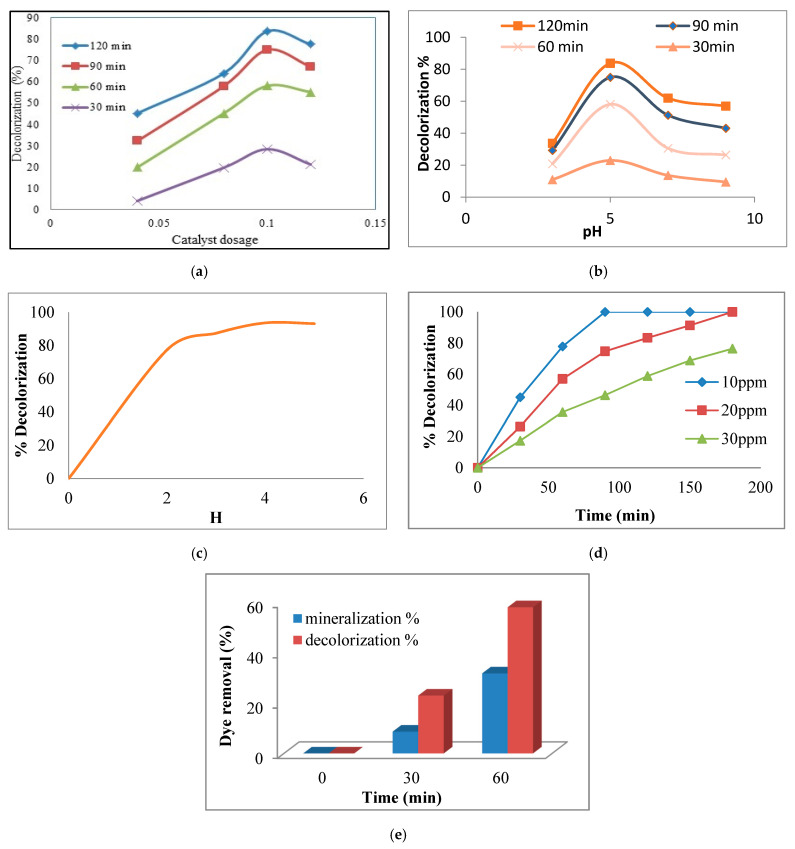
(**a**) Effect of catalyst dosage on decolorization efficiency at different HRTs (dye concentration of 20 ppm and pH 5); (**b**) the effect of pH on decolorization of RR198 at different HRTs (the dye concentration of 20 ppm and catalyst dosage 0.10 g/L); (**c**) decolorization plot on different amount of H [H_2_O_2_/Dye] (dye concentration of 20 ppm, catalyst dosage 0.10 g/L, and pH of 5); (**d**) decolorization of RR198 versus HRT for different dye concentration (catalyst dosage of 0.10 g/L and pH of 5); (**e**) decolorization and mineralization of RR198 at different HRTs (catalyst dosage of 0.10 g/L, dye concentration 20 ppm, and pH of 5).

As seen in Figure 7a, decolorization performance of synthetic wastewater contaminated with 20 ppm RR198 dye in the presence of 0.04, 0.08, 0.1, and 0.12 g/L of the ZnO-Nd nanoparticles was 45.2%, 59.9%, 83.7%, and 77.6%, respectively. Figure 7a shows that by increasing the nano-photocatalyst concentration and hydraulic retention time (HRT), the dye removal efficiency increased. The results illustrated that under the nano-photocatalyst concentration of 0.04 g/L and the HRT of 30 min, the dye removal efficiency was only 6%. Increasing the concentration of nano-photocatalyst to 0.1 g/L leads to increasing the dye removal efficiency of synthetic wastewater up to 25%. By increasing the concentration of nano-photocatalyst to 0.12 g/L, there was no increase in the efficiency of dye removal from wastewater. Increasing the concentration of nano-photocatalysts to 0.1 g/L could increase the production of OH^•^ and O_2_^•^ radicals, resulting in increasing the removal efficiency of RR198 dye from synthetic wastewater. Increasing the concentration to more than 0.1 g/L could increase the turbidity of synthetic wastewater and reduce the penetration of UV light into it [39,40].

Reducing the penetration of UV light means reducing photocatalytic reactions, resulting in reducing the efficiency of dye removal from synthetic wastewater. Therefore, increasing the concentration of the nano-photocatalyst to 0.12 g/L decreased the dye removal efficiency. By using 0.1 g/L of nano-photocatalyst and varying HRT from 30 min to 120 min, the dye removal efficiency reached 85% (Figure 7a). Furthermore, once the ZnO-Nd nano-photocatalysts were added to the reactor as suspended particles, a huge surface for photocatalytic reactions was provided. Therefore, the wastewater after treatment contains these suspended nanoparticles which must be removed before being released into the environment. Since the nanoparticles cannot be easily settled by a simple settling tank, a chemical or electrochemical coagulation process is suggested to remove them from the effluent of the reactors.

#### 3.2.2. Effect of pH

pH is one of the factors affecting photochemical reactions. The results showed that 33%, 83%, 62%, and 58% of RR198 dye was removed with pHs 3, 5, 7, and 9, respectively. It was also found that increasing the pH from 3 to 5 in HRT of 30 min could increase the dye removal efficiency from 33% to 83% (Figure 7b). Increasing the pH from 5 to 9 reduced the dye removal performance from 83% to 58%. A similar trend was observed in HRTs of 60, 90, and 120 min. In higher pHs, the negative charge increases on the surface of the catalyst, and, as the dye has a negative charge, electrostatic repulsive energy is created which causes the reduction in decolorization performance [23]. Furthermore, the decomposition of ZnO nanoparticles increased under acidic pH, which can be considered the reason for low dye removal performance under a pH of 3 [41]. Based on the abovementioned reasons, the pH of 5 was selected as the best pH for photocatalytic oxidation of RR198 dye. Other experiments in this study with various HRTs were carried out at a pH of 5. By increasing the HRT from 30 min to 120 min, initial dye concentration of 10 ppm greatly increased the dye removal efficiency up to 100%. Therefore, at a pH of 5 and an HRT of 120 min, 83% of the dye was removed (Figure 7b). Decolorization potential of ZnO:Nd and pure ZnO was compared with each other at the pH of 5. The results illustrate that the decolorization ability of ZnO increased about 11% when doped with Nd to form ZnO:Nd.

#### 3.2.3. Effect of Hydrogen Peroxide (H_2_O_2_)

H_2_O_2_ has different effects on photocatalytic degradation. These effects depend on the concentration and the nature of the dye. Hydroxyl radicals were generated in two ways. First they were produced through ZnO reduction on the conduction band of the photocatalyst, and secondly due to self-decomposition by light irradiation [42]. Figure 7c shows the effect of H_2_O_2_ concentration on dye removal. In this figure, the ratio of H_2_O_2_/dye is used to better understand the H_2_O_2_ effects on RR198 dye removal. This ratio was calculated by $H=\left[H_{2}O_{2}\right]/\left[\mathrm{dye}\right]$. The values of this parameter changed from 0 to 5. With the increase in the amount of H from 0 to 2, the decolorization sharply increased. When the experiments were carried out under an H of more than two, the decolorization performance was approximately stable at 90%. When the amount of H_2_O_2_ concentration goes beyond the optimal amount, H_2_O_2_ acts as a trap of valence band holes and hydroxyl radicals [43]. The addition of hydrogen peroxide could promote degradation since it can be reduced by conduction band electrons and the superoxide radical anions to yield hydroxyl radicals. However, hydrogen peroxide can also be directly oxidized by the valence band holes and wasted to oxygen and protons, reacting with hydroxyl radicals. HO_2_^o^ radicals created during chain reactions were oxidizers (Equations (8)–(10)). However, their oxidizing potential was much less than the oxidizing potential of hydroxyl radicals. Thus, extra hydrogen peroxide radicals had a positive impact on decolorization.

(8) $$H_{2}O_{2}+2h_{\mathrm{VB}}^{+}\to O_{2}+2H^{+}$$

(9) $$H_{2}O_{2}+{\mathrm{OH}}^{{}^{\circ}}\to {\mathrm{HO}}_{2}^{{}^{\circ}}+H_{2}O$$

(10) $${\mathrm{HO}}_{2}^{{}^{\circ}}+{\mathrm{OH}}^{{}^{\circ}}\to H_{2}O+O_{2}$$

During the degradation by ZnO-Nd, experiments showed that first the azo dyes’ bonds were degraded and then converted to simple organic compounds [44]. The following chemical equations also show the pathway of dye degradation using ZnO-Nd nanoparticles (Equations (11)–(19)) [44].

(11) $$\mathrm{ZnO}+\mathrm{hv}\to e_{{\mathrm{CB}}^{-}}+h_{{\mathrm{VB}}^{+}}$$

(12) $${\mathrm{Nd}}^{3+}+e_{{\mathrm{CB}}^{-}}\to {\mathrm{Nd}}^{2+}$$

(13) $${\mathrm{Nd}}^{2+}+O_{2}\to {\mathrm{Nd}}^{3+}+O_{2}^{-}$$

(14) $$h_{{\mathrm{VB}}^{+}}+H_{2}O\to H^{+}+{\mathrm{OH}}^{{}^{\circ}}$$

(15) $$h_{{\mathrm{VB}}^{+}}+{\mathrm{OH}}^{-}\to {\mathrm{OH}}^{{}^{\circ}}$$

(16) $$e_{{\mathrm{CB}}^{-}}+O_{2}\to O_{2}^{{}^{\circ}-}$$

(17) $$O_{2}^{{}^{\circ}-}+H^{+}\to {\mathrm{HO}}_{2}^{{}^{\circ}}$$

(18) $$\mathrm{Dye}+{\mathrm{OH}}^{{}^{\circ}}\to \mathrm{Degradation}\;\mathrm{products}$$

(19) $$\mathrm{Dye}+{\;e}_{{\mathrm{CB}}^{-}}\to \mathrm{Reduction}\;\mathrm{products}$$

#### 3.2.4. Effect of Initial Dye Concentration

The effect of primary dye concentration on photocatalyst efficiency was evaluated in the three concentrations of 10, 20, and 30 ppm. The results showed that the increase of dye concentration from 10 to 30 ppm reduced photocatalytic dye removal (Figure 7d). A hypothesis for the reduction in RR198 dye removal performance from synthetic wastewater within the photocatalytic oxidation may be the reduction in the penetration of UV light through the wastewater at higher dye concentrations. Seven experiments were done with synthetic wastewater contaminated with different concentrations of RR198 dye (10, 20, 30, 40, 50, 60, 70, 80, 90, and 100 ppm). Then the absorption of each one was measured between the wavelengths of 200 and 800 nm (Figure 2). The results showed that light absorption by the photocatalyst increased, so photocatalytic decolorization efficiency was promoted. However, as the dye concentration increased, decolorization decreased. This is because, by increasing dye concentration, more dye molecules were adsorbed on the catalyst surface. Hence, less light can reach the catalyst surface, and a less active catalyst site is available to produce hydroxyl. On the other hand, less light can penetrate through the solution and reach the catalyst surface by increasing dye concentration. This proved that the penetration of light into synthetic wastewater can be limited by increasing the dye concentration. On the other hand, at high concentrations of RR198 dye, the surface of the catalyst absorbs more dye molecules, and the number of reachable sites decreases. These factors prevent the formation of hydroxyl and superoxide.

A set of the experiment was done under catalyst dosage of 0.10 g/L and pH of 5 with initial dye concentrations of 10, 20, and 30 ppm. After only 90 min nearly 100% of decolorization appeared when the falling-film photocatalytic reactor was performed under an initial dye concentration of 10 ppm. Approximately 175 min are required to reach 100% decolorization under an initial dye concentration of as much as 20 ppm. The results showed that a higher initial dye concentration means a higher needed HRT to remove the dye from synthetic wastewater.

### 3.3. Mineralization

There is a difference between the decolorization and the mineralization process. Under the decolorization process, the organic dye molecule may break into another colorless organic matter. In this situation, although the color has disappeared, the TOC of water is still high. In the mineralization process, the organic dye will be decomposed into inorganic matters such as H_2_O and CO_2_. In the mineralization process, not only can the color of the water disappear, but the water TOC is also reduced. To measure the amount of mineralization, RR198 analysis of TOC was carried out and the results are shown in Figure 7e. TOC elimination does not comply with the amount of decolorization. It was almost 31.79% in 120 min, while a decolorization of 58.10% was measured in the same HRT. These results show that the azo group N=N breaks faster compared with the aromatic ring. Thus, the speed of decolorization is faster than mineralization [45].

### 3.4. Study of Decolorization Reaction Kinetic

For checking the kinetics of the photocatalyst, three concentrations of the dye RR198 were prepared (10, 20, 30 ppm). They were irradiated for 180 min via UV light in the falling-film photoreactor. The Langmuir-Hinshelwood equation was used for kinetic modeling. The Equation (20) is stated as follows:

(20) $$r=\frac{-\mathrm{dc}}{\mathrm{dt}}=\frac{k_{r}k_{\mathrm{ad}}c}{1+k_{\mathrm{ad}}c}\;$$

where k_r_ is the constant of reaction rate and k_ad_ is the adsorption equilibrium constant. The results of this study show that the amount of adsorption is small so that K_ad_C is smaller than 1 in this process, so it can be ignored. Thus, the above equation is changed to the linear Equation (21).

(21) $$r=\frac{-\mathrm{dc}}{\mathrm{dt}}=k_{r}k_{\mathrm{ad}}c$$

Integrating both sides of the above Equation (22), the following expression is obtained:

(22) $$\ln(\frac{c_{0}}{c})=k_{r}k_{\mathrm{ad}}t=k_{\mathrm{ap}}t$$

**Figure 8:**
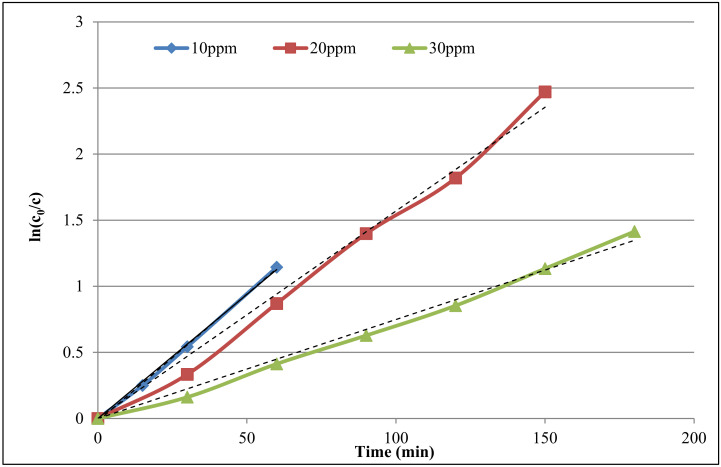
Kinetic study of RR 198 decolorization with different dye concentrations (catalyst dosage 0.10 g/L and pH 5, as well as C_0_ and C, are the initial and final concentrations).

If ln(c_0_/c) is plotted versus time, the result will be a line. The slope of this line gives K_ap_. (Figure 8) The parameters k and R^2^ (correlation constant) of the decolorization process are shown in Table 1 [46].

### 3.5. Response Surface Methodology (RMS) Analysis

#### 3.5.1. Analysis of Variance (ANOVA) and Statistical Analysis

Table S1 (in the section of Supplementary Materials) shows that the developed model to predict the RR198 decolorization from the synthetic wastewater matches with the experimental data in Table 1. The dye decolorization using the falling-film photocatalytic reactor was modeled based on the RSM method to obtain Equation (23). This equation can be used to predict the performance of the RR198 dye in a falling-film photocatalytic reactor. The weakness of these equations is that they can only predict the performance of the dye decolorization at an HRT of 60 min. The final Equation in terms of coded factors is:

(23) $$R1=55+14.01A+6.85B-15.82C+1.51\left(\mathrm{AB}\right)-8.51\left(\mathrm{AC}\right)-1.35\left(\mathrm{BC}\right)-12.89A^{2}-17.91B^{2}+4.23C^{2}$$

where R1 is the dye removal performance at HRT of 60 min in percentage, A is catalyst dosage in mg/L, B is the synthetic wastewater pH, and C is the initial dye concentration in ppm. The main effect of each variable was estimated as the difference between both averages of measurements made at the high level (+1) and at the low level (−1) of that nutritional factor. Equation (23) was indicated based on the variance of analysis. The values of F and Prob > F are the main indices of Equation (23), which indicate their importance and capability. A Prob > F less than 0.05 shows that the equations are appropriate and an accurate prediction of the dye decolorization. It was less important if its value was bigger than 0.1. ANOVA was studied for the decolorization process of RR198 dye in the falling-film photocatalytic reactor. This showed that the investigated parameters were very important. Based on the small amount of F (16.98) and a very small amount of Prob > F (0.0001), it was concluded that the provided equations were appropriate for the expression of the experimental data. The results showed that the catalyst dosage (A), the synthetic wastewater pH (B), the initial concentration (C), the product of catalyst dosage and initial dye concentration (A × C), the square of catalyst dosage (A^2^), and the square of the synthetic wastewater pH (B^2^) were important parameters of the model. These important parameters have Prob > F less than 0.05. Some of the results showed that certain variables such as catalyst dosage and pH have a synergistic effect on dye removal performance and some others such as dye concentration have an antagonistic effect on the dye removal performance (see Table S2 in Supplementary Materials). Dye concentration had the largest regression coefficient, which shows that it had the most important antagonistic effect on the dye decolorization performance. The most important synergistic factor is also the catalyst dosage. When the catalyst dosage increases, decolorization performance increases too. Such behavior has been observed in the other studies [47].

#### 3.5.2. Optimization of Degradation of ZnO-Nd under UV Irradiation Using RSM

**Figure 9:**
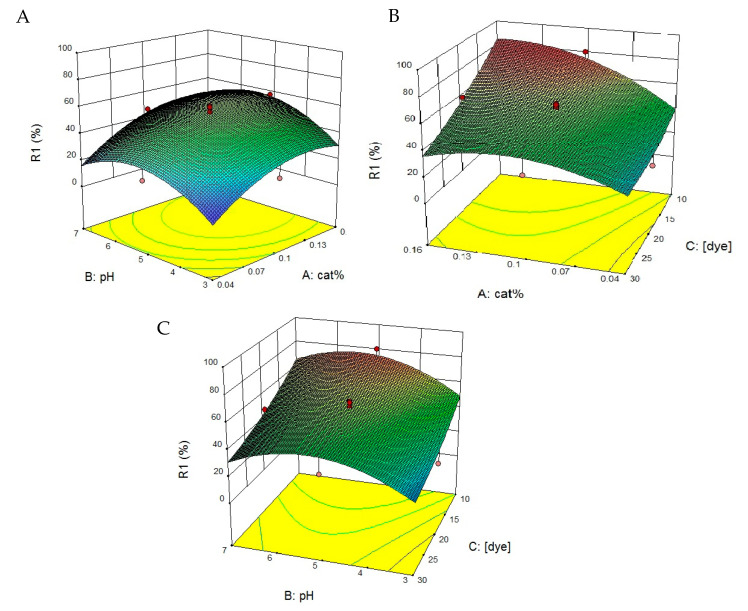
Response surface plots showing the interaction of independent variables on each other for degradation of RR198 by ZnO-Nd under UV light ((**A**) catalyst dosage and pH, (**B**) catalyst dosage and initial dye concentration, and (**C**) pH and initial dye concentration).

To show the destruction of RR198 dye by ZnO-Nd, 3-D plots of response surface were drawn. In each design, two parameters were changed while the third parameter was kept constant. Response surface plots are shown in Figure 9. This study showed that catalyst dosage and pH had a prominent effect on RR198 decolorization. By increasing the amount of catalyst from the optimum level, the amount of dye was reduced due to the prevention of light radiation. Interactive interaction (%cat × [dye]) was negative according to the regression coefficient (see Table S3 in Supplementary Materials). This shows that active sites (which increase with the increase in catalyst dose) were less than the number of dye molecules that add to the synthetic wastewater with increasing concentration. Thus, the antagonistic effect was dominant on the synergism in the interactive interaction. Finally, the best response predicted was the removal of an 81.85% dye at an HRT of 60 min.

Figure 9A shows that the best results for the removal of RR198 dye obtained by increasing the catalyst dosage in pH of 5. Also, by increasing in catalyst dosage and dye concentration, the highest amount of RR198 dye removal was obtained (Figure 9B). Furthermore, a high amount of RR198 dye concentration under pH between 4 and 5.5 was the best condition for effective dye removal (Figure 9C). 

### 3.6. Cost Analysis

Since photocatalysis of an organic contaminant in a liquid solution depends on electrical energy, electrical energy can reveal some practical costs. The Evaluation of Electrical Energy per Order (EEO) is defined as the kilowatt-hour electrical energy needed for the removal of 90% of contaminant’s concentration in one cubic meter of contaminated water. EEO (kWh/m^3^) can be calculated by Equation (24) at each order [45,48].

(24) $$\mathrm{EEO}=\frac{38.4P}{\mathrm{VK}}$$

where P is the rated power of the AOP system in kW, K is the pseudo-first-order rate constant for the decay of the pollutant concentration in min^−1^, V is the volume of the water in the reactor in L. The value of EEO for each one of the concentrations of 10, 20, and 30 ppm was calculated as 40.851, 48.917, and 102.4 kWh/m^3^, respectively. Since the global average of each kW of electrical energy is around USD 0.1319, the cost of decolorization of each cubic meter of contaminated water with RR198 dye is estimated to be as much as USD 5.38, 6.45, and 13.5 for wastewater with the dye concentrations of 10, 20, and 30 ppm, respectively. On average, the wastewater decolorization of wastewater contaminated with the RR198 dye with a concentration between 10 and 30 ppm is USD 8.44.

## 4. Conclusions

In this study, the effects on photocatalyst dosage, pH value, the ratio of H_2_O_2_/dye, and initial dye concentration were investigated. Under a pH of more than 5, the negative charge increases on the surface of ZnO-Nd nano photocatalyst, and electrostatic repulsive energy was created which causes the reduction in decolorization performance. The best pH value to conduct the falling-film photocatalyst reactor was five. Under pH values less than five, the decomposition of ZnO nanoparticles increases. Therefore, a pH of five was the best pH to decolorize synthetic wastewater contaminated with RR198 dye. Using the ratio of H_2_O_2_/dye at two showed to be the best ratio to obtain the highest dye removal performance. Increasing the initial dye concentration had a deteriorative effect on the RR198 dye removal from the synthetic wastewater due to (a) the reduction of UV penetration into the synthetic wastewater and (b) decreasing the number of reachable sites on the surface of photocatalysis nanoparticles that can prevent the formation of hydroxyl and superoxide. In this study, two equations were developed that can be used to predict the performance of the falling-film photocatalyst reactor under various operating conditions such as pH, the initial dye concentration, and the catalyst dosage. The falling-film photocatalyst reactor is an innovative and appropriate reactor type to obtain a high dye removal performance from water.

## Figures and Tables

**Table 1:** Reaction rate constants and correlation constants.

Dye Concentration (ppm)	k (min^−1^)	R^2^
10	0.0188	0.9979
20	0.0157	0.9903
30	0.0075	0.9912

## Supplementary Materials

The following are available online at https://www.mdpi.com/article/10.3390/toxics9100254/s1. Table S1: Regression analysis and significance of the components in the quadratic model for RR198 degradation by ZnO-Nd; Table S2: Analysis of variance of the developed quadratic model for RR198 degradation by ZnO-Nd in presence of UV light; Table S3: Coefficient of regression and standard errors.
