# Supplementary material for: Application of ZnO-Nd Nano-Photocatalyst for the Reactive Red 198 Dye Decolorization in the Falling-Film Photocatalytic Reactor
Source: Toxics. 2021 Oct 8;9(10):254. doi: 10.3390/toxics9100254 (PMC8540491; doi:10.3390/toxics9100254)
Supplement: Supplementary file 1 [file toxics-09-00254-s001.zip › toxics-1395937-supplementary.pdf]

toxics MDPI

Supplementary Materials: Application of ZnO-Nd Nano-Photocatalyst for the Reactive Red 198 Dye Decolorization in the Falling-Film Photocatalytic Reactor

Fatemeh Biglar, Amirreza Talaiekhozani *, Farham Aminsharei, Junboum Park, Anahita Barghi and Shahabaldin Rezania *

Table S1. Regression analysis and significance of the components in the quadratic model for RR198 degradation by ZnO-Nd.

Std	Run	Factor 1<br>A: Catalyst dosage	Factor 2<br>B: pH	Factor 3<br>C: [Initial dye concentration]	Response 1<br>%R1
8	1	0.16	7	30	25.72
7	2	0.04	7	30	14.64
9	3	0.04	5	20	19.79
14	4	0.1	5	30	31.37
15	5	0.1	5	20	56.13
4	6	0.16	7	10	73.24
2	7	0.16	3	10	56.32
3	8	0.04	7	10	27.15
17	9	0.1	5	20	60.3
18	10	0.1	5	20	60.46
12	11	0.1	7	20	46.51
16	12	0.1	5	20	56.03
6	13	0.16	3	30	15.17
13	14	0.1	5	10	80.3
19	15	0.1	5	20	59.67
1	16	0.04	3	10	17.23
10	17	0.16	5	20	57.63
5	18	0.04	3	30	9.18
11	19	0.1	3	20	20.88

Table S2. Analysis of variance of the developed quadratic model for RR198 degradation by ZnO-Nd in presence of UV light.

Source	Sum of Squares	df	Mean Square	F Value	p-value Prob > F
**Model**	8488.35	9	943.15	16.98	0.0001
**A-cat%**	1962.52	1	1962.52	35.32	0.0002
**B-pH**	468.95	1	468.95	8.44	0.0174
**C-[dye]**	2501.46	1	2501.46	45.02	< 0.0001
**AB**	18.27	1	18.27	0.33	0.5804
**AC**	579.87	1	579.87	10.44	0.0103
**BC**	14.66	1	14.66	0.26	0.6198
**A^2**	454.33	1	454.33	8.18	0.0188
**B^2**	876.44	1	876.44	15.78	0.0032
**C^2**	48.90	1	48.90	0.88	0.3727

Table S3. Coefficient of regression and standard errors.

Factor	Coefficient Estimate	Standard Error
Intercept	55.45	2.73
A-cat%	14.01	2.36
B-pH	6.85	2.36
C-[dye]	−15.82	2.36
AB	1.51	2.64
AC	−8.51	2.64
BC	−1.35	2.64
A^2	−12.89	4.51
B^2	−17.91	4.51
C^2	4.23	4.51
